# Interactive Effects of Temperature, Water Regime, and [CO_2_] on Wheats with Different Heat Susceptibilities

**DOI:** 10.3390/plants13060830

**Published:** 2024-03-13

**Authors:** Rong Zhou, Benita Hyldgaard, Lamis Abdelhakim, Thayna Mendanha, Steven Driever, Davide Cammarano, Eva Rosenqvist, Carl-Otto Ottosen

**Affiliations:** 1 College of Horticulture, Nanjing Agricultural University, Nanjing 210095, China; 2 Department of Food Science, Aarhus University, DK-8200 Aarhus, Denmark; behy@seges.dk (B.H.); lamisabdelhakim@gmail.com (L.A.); tm@food.au.dk (T.M.); coo@food.au.dk (C.-O.O.); 3 Centre for Crop Systems Analysis, Wageningen University, Bornsesteeg 48, 6708 PE Wageningen, The Netherlands; steven.driever@wur.nl; 4 Department of Agroecology, iClimate, CBIO, Aarhus University, 8830 Tjele, Denmark; davide.cammarano@agro.au.dk; 5 Department of Plant and Environmental Sciences, University of Copenhagen, DK-2630 Taastrup, Denmark; ero@plen.ku.dk

**Keywords:** wheat, physiological response, elevated CO_2_ concentration, reduced watering, increased temperature

## Abstract

Plants’ response to single environmental changes can be highly distinct from the response to multiple changes. The effects of a single environmental factor on wheat growth have been well documented. However, the interactive influences of multiple factors on different wheat genotypes need further investigation. Here, treatments of three important growth factors, namely water regime, temperature, and CO_2_ concentration ([CO_2_]), were applied to compare the response of two wheat genotypes with different heat sensitivities. The temperature response curves showed that both genotypes showed more variations at elevated [CO_2_] (e[CO_2_]) than ambient [CO_2_] (a[CO_2_]) when the plants were treated under different water regimes and temperatures. This corresponded to the results of water use efficiency at the leaf level. At e[CO_2_], heat-tolerant ‘Gladius’ showed a higher net photosynthetic rate (P_n_), while heat-susceptible ‘Paragon’ had a lower P_n_ at reduced water, as compared with full water availability. The temperature optimum for photosynthesis in wheat was increased when the growth temperature was high, while the leaf carbon/nitrogen was increased via a reduced water regime. Generally, water regime, temperature and [CO_2_] have significant interactive effects on both wheat genotypes. Two wheat genotypes showed different physiological responses to different combinations of environmental factors. Our investigation concerning the interactions of multi-environmental factors on wheat will benefit the future wheat climate-response study.

## 1. Introduction

The effect of adverse climate on terrestrial ecosystems has been an ongoing source of concern over the past decades. Anthropogenic activities have led to an increase in the atmospheric CO_2_ concentration ([CO_2_]), ascribed to be the driving force of climate change [1]. Future agriculture production is expected to face not only rising [CO_2_] levels but also a combination of other factors, such as warmer global temperatures, drought stress, and an increase in extreme climatic events. Crop yields have already been impacted by climate change as indicated by a global annual yield decrease in rice and wheat (0.3% and 0.9%, respectively) due to climate change reported by Ray et al. (2019) [2]. In Europe, yields of all non-tropical crops have decreased (6.3–21.2%) due to climate change [2]. Despite the continuous genetic progress, climate change may be the primary cause behind the decline in the yield growth trend for cereals in Europe [3,4,5]. 

Even though the response of plants to individual stresses, such as high temperature, drought, and elevated [CO_2_] (e[CO_2_]), are extensively discussed in the literature, relatively little is known about the physiological response of plants to the interaction of those factors. Multifactor stresses are complex, and a positive plastic response to a single stress may play an antagonistic role when other stresses are added [6]. For instance, stomatal regulation contrasts upon its response to drought and heat stresses: while drought closes stomata to prevent water loss, heat stress increases stomatal conductance to cool the leaves by transpiration. 

Photosynthesis is one of the highly thermo-sensitive processes in plants, which can be affected by temperature changes in many ways [7,8]. Meanwhile, the water deficit can affect the photosynthesis of wheat in various degrees depending on genotypes [9]. Overlapping changes in temperature and water might intensify the injuries experienced by the photosynthetic apparatus [10]. Still, plants can acclimate to stressful conditions. For example, wheat and rice are able to acclimate their photosynthesis to temperature and elevated [CO_2_] during the growing season [11]. The acclimation of photosynthesis in a valuable crop such as wheat is crucial to study, since the inhibition of photosynthesis is directly associated with a decreased yield and optimizing photosynthesis serves to maximize carbon gain [12]. 

Water use efficiency (WUE; g kg^−1^) is a key indicator of drought tolerance. Crop management increased the yield, which also resulted in high WUE [13], indicating the positive relation between crop yield and WUE. Numerous authors have demonstrated substantial differences between the WUE values of cereal species [14,15,16] but have also emphasized the fact that changes in WUE are especially important if the water supply to the plants is limited [17]. A synergetic response of WUE is usually reported to the combination of drought and e[CO_2_] on plants [6,18]. Similarly, while higher temperatures increase respiration more than photosynthesis, e[CO_2_] alters the ratio of [CO_2_]/[O_2_] towards [CO_2_], favoring Rubisco carboxylation over oxygenation, hence alleviating carbon loss due to photorespiration under heat stress conditions [19]. Moreover, carbon and nitrogen metabolism of plants under drought are strongly interrelated [20]. Additional information is required about actual crop evapotranspiration and water use levels when plants suffer different levels of stress during different growth stages [21]. 

Plants experience oxidative damage due to the overaccumulation of reactive oxygen species (ROS) and shortage in the production of enzymatic and non-enzymatic antioxidants under drought and heat stress. For instance, carotenoids play a crucial role in antioxidant defense systems, as they can serve as antioxidants in crops in adverse environments. Plants grown in e[CO_2_] have been reported to up-regulate the antioxidant defense by the excessive carbohydrate production and the decrease in photorespiration, therefore attenuating oxidative stress [22,23]. In addition, under heat, drought, and their combination, wheat yield reduction is reported to be alleviated by e[CO_2_] [24], due to an increase in photosynthesis [25]. Yet, multigenerational exposure to e[CO_2_] aggravated grain quality reduction by reducing N, K, Ca, protein, and total amino-acid concentration in wheat grains [24]. Clearly, the effects of e[CO_2_] on wheat being subjected to temperature changes and water regimes are complicated and require further exploration. 

In this study, two wheat cultivars with different levels of susceptibility to heat were exposed to two temperature levels during the earlier vegetative phase with full and reduced watering under ambient [CO_2_] (a[CO_2_]) and e[CO_2_]. Photosynthetic temperature response curves, WUE, optimum temperature, integrated carbon gain, leaf area, and the C/N ratio were investigated. We aimed to clarify the interactive effects of three factors, namely temperature, water, and [CO_2_], on wheat physiology. We hypothesized that (1) the detrimental effect of reduced water, temperature changes, and their combination on plant photosynthesis could be alleviated by e[CO_2_], as it allowed photosynthetic rates to be maintained under non-optimum conditions; and (2) the physiological response of the two wheat genotypes to temperature changes, water regime, and [CO_2_] could differ, as heat-tolerant wheat showed high resilience. Studies on multifactor stresses in cereals will contribute to an increase in the understanding of plant responses under realistic field situations and can improve current stress management and CO_2_ fertilization strategies. The inclusion of different genotypes in such studies can add to the understanding of crucial adaptation traits to environmental changes and the pursuit of engineering a better stress-tolerant germplasm.

## 2. Materials and Methods

### 2.1. Plant Material and Experimental Treatments

Two commercial spring wheat cultivars (*Triticum aestivum* L.) were studied: heat-tolerant ‘Gladius’ from Australia and heat-susceptible ‘Paragon’ from the UK [26]. One seed was sown in 0.6 L plastic pots. The pots were filled with commercial peat substrate (Pindstrup Færdigblanding 2, Pindstrup Mosebrug A/S, Ryomgaard, Denmark). The seedlings were cultivated in a greenhouse for 14 days during the summer of the year 2015, at the Department of Food Science, Aarhus University, Aarslev, DK (55.30 N, 10.44 E). Seedlings were grown at ambient CO_2_ (400 ppm), with an average air temperature of 24 °C. Supplementary light (high-pressure sodium lamps, SON-T Agro, 600 W, Philips, Eindhoven, The Netherlands) was provided whenever the natural photosynthetic photon flux density (PPFD) reached below 150 µmol m^−2^ s^−1^. Once the seeds sprouted, pots were fertigated daily by flooding the greenhouse table with a nutrient solution (N:P:K of 190:35:275 ppm, pH 5.8, electrical conductivity of 1.9 mS m^−1^). 

**Figure 1:**
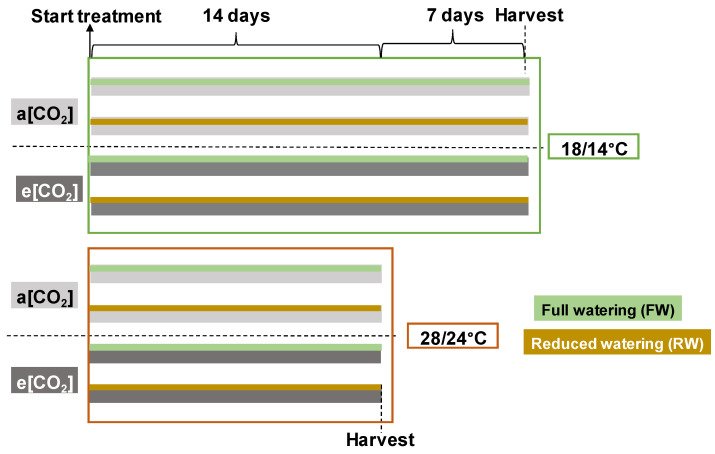
Illustration of the eight established treatments. To ensure plant harvest at the same developmental stage, the plants grown at 18/14 °C were harvested one week after those grown at 28/24 °C.

Vigorous uniform plants at growth stage Zadoks 12–13 [27] were moved into two controlled climate chambers in two batches separated in time due to space limitations. During the first batch, plants were grown at 400 ppm CO_2_ concentration (a[CO_2_]), and during the second round, at 800 ppm CO_2_ concentration (e[CO_2_]). There were four environmental settings in the climate chambers for each CO_2_ concentration: (1) 18/14°C air temperature and full watering (FW) in which pots were kept at 100% field capacity as control; (2) 28/24 °C air temperature and FW in which pots were kept at 100% field capacity; (3) 18/14 °C air temperature and reduced watering (RW) by keeping pots at 50% field capacity; and (4) 28/24 °C air temperature and RW by keeping pots at 50% field capacity. In total, eight treatments were established, as shown in Figure 1. Values of air relative humidity (RH) were adjusted to 45/47% at 18/14 °C and 73/67% at 28/24 °C to ensure a vapor deficit temperature (VPD) of approximately 1 kPa. For all treatments, the light level was set at 280 µmol m^−2^ s^−1^ (Fionia Lighting LED FL300 sunlight, Søndersø, Denmark) at plant canopy level supplied in a 14/10 h day/night photoperiod regime corresponding to a daily light integral (DLI) of 14.11 mol m^−2^ day^−1^. The water regime was imposed using drought spotter units (Phenospex, Heerlen, The Netherlands) installed at each chamber. Pots were assigned to the scales in a completely randomized design (n = 6). The treatments with 18/14 °C and 28/24 °C lasted 21 days and 14 days, respectively, in order to obtain harvest plants at the same developmental stage. 

### 2.2. Leaf Area and Carbon/Nitrogen (C/N)

Plants grown in a control chamber (18/14 °C) were harvested seven days later (21 days) than plants grown in a warm chamber (14 days) in an attempt to end the experiment with all treatments at a similar developmental stage. Leaf area (LA) was determined using a LI-3100C area meter (Li-Cor, Lincoln, NE, USA). A 3–4 cm piece of primary stem (3 cm above soil surface) was also harvested for C/N determination. C/N determination was performed at Aberystwyth University (Aberystwyth, Wales, UK) via an elemental analysis (Elementar Vario Max Q CN analyser) [28]. There were six replicates. 

### 2.3. Leaf Photosynthetic Pigments

The leaf used for photosynthesis measurements was sampled after 14 days of the treatments, weighted, and immediately stored at −80 °C for pigment analysis. Leaf samples were freeze-dried, weighed, and milled in a mixer mill (MM200, Retsch Inc., Haan, Germany), using a steel ball. Photosynthetic pigments were extracted with 96% Ethanol overnight at room temperature. Contents of pigments, including chlorophyll a (Chl a), chlorophyll b (Chl b), and carotenoids (Car), were determined spectrophotometrically (Shimadzu UV-1700, Kyoto, Japan), according to the method of Lichtenthaler (1987) [29]. The ratios of chlorophylls a:b (Chl a:b) and total chlorophylls to carotenoids (Chl:Car) were calculated. There were six replicates.

### 2.4. Photosynthetic Temperature Response Curves 

Photosynthetic temperature response curves (A/T) were performed after 14 days of treatment, using a portable gas exchange fluorescence system (CIRAS-2, PP systems, Amesbury, MA, USA). The last young fully developed leaf was affixed in a 1.7 cm^2^ leaf cuvette with a 195 mL min^−1^ flow rate and 1500 µmol photons m^−2^ s^−1^ PPFD controlled by an LED light unit. The CO_2_ concentration of the cuvette was set to 400 and 800 ppm for the plants grown under a[CO_2_] and e[CO_2_], respectively. The CIRAS cuvette and the plant were placed in a controlled climate cabinet during the measurements, and the temperature in the cabinet was adjusted according to the following set points: 15 °C, 18 °C, 25 °C, 30 °C, 35 °C, and 40 °C. Once the steady leaf temperature was reached, the gas exchange rates were recorded. A moist cloth was placed around the grid of the water vapor equilibrator of the gas analyzer to increase relative humidity when needed and maintain VPD around 1 kPa. There were three replicates.

### 2.5. Leaf Water Use Efficiency

The water use efficiency at the leaf level (WUE_leaf_) was calculated by P_n_/E (net photosynthetic rate and transpiration). The optimum temperature (T_opt_) was calculated as the vertex of a hyperbola fitted for each A/T response curve. The integrated carbon gain (∫ P_n_) was calculated by the sum of the trapezoid area of each temperature point. There were three replicates.

(1) $$\int P_{n}=\left(P_{n1}+P_{n2}\right)\times \frac{\left(T_{2}-T_{1}\right)}{2}$$

where T_1_ and T_2_ indicate two temperatures, while P_n1_ and P_n2_ indicate the net photosynthetic rate at the two temperatures. 

### 2.6. Data Analysis

Statistical analyses were performed using R open-source statistical computing software (Version 3.4.3, The R Foundation, Vienna, Austria). Data were checked for variance homogeneity and normal distribution before statistical analysis. Means were compared using a pairwise comparison procedure adjusted with Bonferroni correction, and the significant difference was indicated by different small letters. The data were analyzed for each cultivar separately. A three-way ANOVA was performed to indicate the effect of [CO_2_], water regime, temperature, and their interaction (CO_2_ × water × temperature), and the level of significance of each factor is indicated as * *p* < 0.05, ** *p* < 0.01, and *** *p* < 0.0001. 

## 3. Results

### 3.1. Leaf Area and C/N

**Figure 2:**
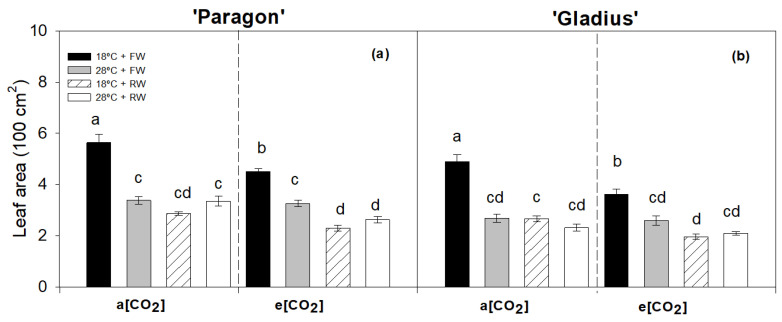
Leaf area of two wheat cultivars, ‘Paragon’ (**a**) and ‘Gladius’ (**b**), for each treatment under a[CO_2_] and e[CO_2_]. Different lowercase letters show significant difference (post hoc adjusted Bonferroni test, *p* < 0.05) between treatments within each panel. ‘Paragon’ data of leaf area were squared root-transformed to pass normality and homogeneity test. Data represent mean values +/− S.E.M. (n = 6).

The e[CO_2_] decreased the leaf area in ‘Paragon’ at 18 °C + FW and 28 °C + RW and in ‘Gladius’ at 18 °C + FW and 18 °C + RW (Figure 2). Reduced water decreased the leaf area in both cultivars at 18 °C regardless of [CO_2_] (Figure 2). Three individual factors had significant effects on leaf area in both cultivars with interaction (Table 1).

**Figure 3:**
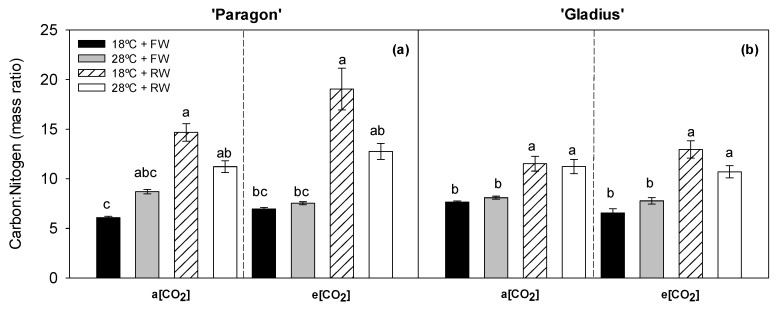
The carbon/nitrogen ratio (C:N ratio) in leaves of two wheat cultivars, ‘Paragon’ (**a**) and ‘Gladius’ (**b**). Different lowercase letters show significant difference (post hoc adjusted Bonferroni test, *p* < 0.05) between treatments within each panel. ‘Gladius’ data were log-transformed to pass the normality and homogeneity test. ‘Paragon’ data did not fulfil the assumption of the parametric method; therefore, they were compared using non-parametric Kruskal–Wallis test. Data represent mean values +/− S.E.M. (n = 6).

In ‘Paragon’, the data for C:N in the leaves did not fulfil the assumption of the parametric method; therefore, the data were compared using a non-parametric Kruskal–Wallis test. The treatments followed a similar trend observed for ‘Gladius’ (Figure 3), as the C:N ratio of leaves was unaffected by either the different [CO_2_] or temperature regimes (Table 1). The RW treatment, however, significantly increased the C:N values under both [CO_2_] for ‘Paragon’ at 18 °C and for ‘Gladius’ at both temperatures (Figure 3). 

### 3.2. Leaf Pigment Content

For ‘Paragon’, Chl a was decreased by e[CO_2_] in plants at RW (Table 2). Reduced water decreased Chl a, Chl b, and Chl:Car in leaves of ‘Paragon’ at e[CO_2_] (Table 2). Increased temperature decreased Chl a content and Chl:Car in leaves of ‘Paragon’ at a[CO_2_] + FW (Table 2). e[CO_2_] increased the Chl a, Chl b, Car content, and Chl:Car, but it decreased Chl a:b in the leaves of ‘Gladius’ at 28 °C + FW (Table 2). Reduced water increased Chl a, Chl b, Car content, and Chl:Car, but it decreased Chl a:b in the leaves of ‘Gladius’ at a[CO_2_] + 28 °C (Table 2). Increased temperature decreased Chl a, Chl b, Car content, and Chl:Car in the leaves of ‘Gladius’ at a[CO_2_] + FW, but it increased Chl b and Car content at a[CO_2_] + RW (Table 2). For both cultivars, all pigments and pigment ratios showed a three-factorial interaction, except for the Car content of ‘Paragon’ (Table 2). 

### 3.3. Photosynthetic Temperature Response

**Figure 4:**
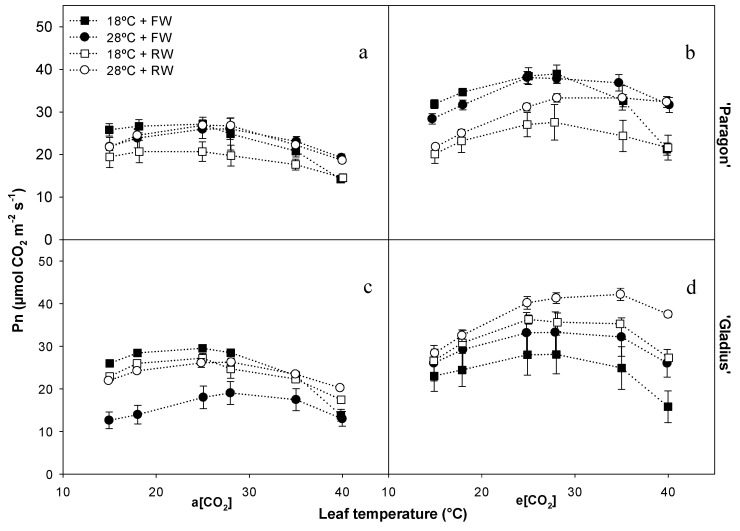
The effects of different treatments in plants grown and measured under a[CO_2_] and e[CO_2_], respectively, on temperature response of net photosynthesis of the wheat cultivars ‘Paragon’ (**a**,**b**) and ‘Gladius’ (**c**,**d**). Data represent mean values +/− S.E.M. (n = 3).

When each point of the temperature curve was compared separately, an interaction between the water regime and [CO_2_] was observed for ‘Paragon’ at temperatures ≤ 25 °C (Table 1). e[CO_2_] alleviated the detrimental effect of reduced water at P_n_ set points 15 °C, 18 °C, and 25 °C when compared to a[CO_2_] (Figure 4a,b and Table 1). For ‘Gladius’, the P_n_ at individual set points of the curves was influenced by the interaction between temperature and [CO_2_] (Table 1). e[CO_2_] alleviated the reduction in P_n_ caused by elevated temperature (28 °C) when compared to a[CO_2_] at all set points, when compared by the parametric method (Figure 4c,d and Table 1).

### 3.4. WUE and Integrated Carbon Gain

**Figure 5:**
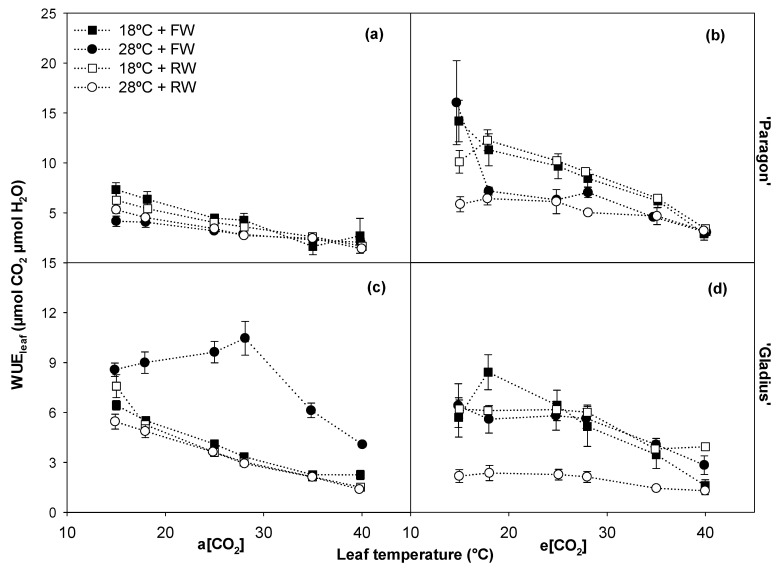
The effects of different treatments under a[CO_2_] and e[CO_2_] on water use efficiency at the leaf level (WUE_leaf_) of two wheat cultivars, ‘Paragon’ (**a**,**b**) and ‘Gladius’ (**c**,**d**). Data represent mean values +/− S.E.M. (n = 3).

When comparing the WUE of the treatments, [CO_2_] and temperature were the factors that consistently influenced most points of the response curve (Table 1). For both cultivars, e[CO_2_] increased WUE as compared with a[CO_2_], while the elevated temperature of 28 °C lowered the parameter in most temperature points as compared with 18 °C (Figure 5). 

**Figure 6:**
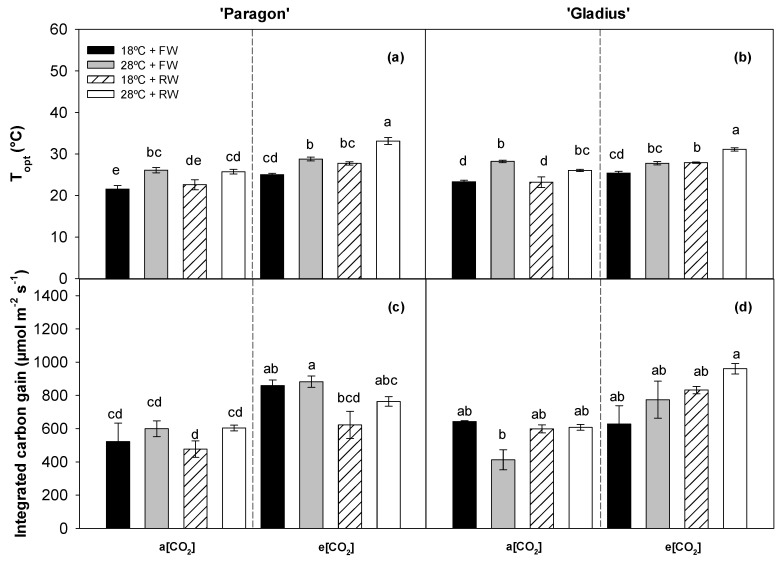
The effects of different treatments under a[CO_2_] and e[CO_2_] on the optimum temperature and the integrated carbon gain of photosynthesis of two wheat cultivars, ‘Paragon’ (**a**,**c**) and ‘Gladius’ (**b**,**d**). Different lowercase letters show significant difference (post hoc adjusted Bonferroni test, *p* < 0.05) between treatments within each panel. ‘Gladius’ data of integrated carbon gain did not fulfil the assumption of the parametric method; therefore, the data were compared using non-parametric Kruskal–Wallis test. Data represent mean values +/− S.E.M. (n = 3).

The e[CO_2_] increased the T_opt_ for ‘Paragon’ grown at 18 °C + FW, 18 °C + RW, and 28 °C + RW and for ‘Gladius’ grown at 18 °C + RW and 28 °C + RW (Figure 6a,b). Reduced water increased the T_opt_ in both cultivars at e[CO_2_], except for ‘Paragon’ grown at 18 °C (Figure 6a,b). A higher temperature increased the T_opt_, except for ‘Paragon’ grown at RW with a[CO_2_] and ‘Gladius’ at FW with e[CO_2_] (Figure 6a,b). Regarding the T_opt_, the only significant interaction noticed was between [CO_2_] and the water regime for both cultivars, even though the individual factor significantly affected T_opt_ (Table 1). The e[CO_2_] increased ∫ P_n_ only in ‘Paragon’ grown at FW as compared with the a[CO_2_] (Figure 6c,d). Accordingly, three individual factors had significant effects on ∫ P_n_ only in ‘Paragon’ without interaction (Table 1).

## 4. Discussions

### 4.1. Physiological Responses of Wheat to Changes on Growth Temperature, Water Regime, and [CO_2_]

Most plants, including wheat, can acclimate their photosynthetic characteristics with continuing increases in growth temperature [7,30]. Even though the general temperature response of photosynthesis is well studied, wheat genotypic variation in photosynthetic thermal acclimation to high temperature needs further investigation [8]. In this study, not only two wheat genotypes with different heat susceptibilities were included but also the interaction between temperature, water regime, and [CO_2_] on wheats were studied (Figure 1). The photosynthetic temperature response curves indicated the effects of temperature changes on photosynthetic CO_2_ fixation. It was found that the P_n_ and WUE_leaf_ curves at different measured temperatures were altered by [CO_2_], with more variation pronounced in two wheat genotypes grown at e[CO_2_] (Figure 4 and Figure 5). This indicated that wheat grown with increased [CO_2_] was more sensitive to the changes in the temperature and water regime. The pigment content analysis presented a three-factorial interaction to chlorophylls *a* and *b* and the ratios between Chl *a:b* and Chl:Car, while the values of the carotenoids content showed two-way interactions (Table 1 and Table 2). Changes in chlorophyll content could partially explain the response of P_n_ in wheat cultivars at different treatments. Moreover, at e[CO_2_], a higher carotenoids content in ‘Paragon’ was induced at 28 °C + FW than that at 18 °C + RW; while that in ‘Gladius’ was higher at 28 °C + RW than that at 18 °C + FW (Table 2). The significant increase in carotenoids content showed that the non-enzyme antioxidant system in wheat actively responded to the environmental changes. In accordance with Berry and Björkman (1980) [7] and Posch et al. (2019) [8], P_n_ increases when the leaf temperature rises, peaking at an T_opt_ and then dropping. However, the T_opt_ can be shifted by the growth environments of the plants. We found that higher growth temperature (28 °C) generally increased T_opt_ as compared with 18 °C (Figure 6). The changes in T_opt_ can be the result of thermal acclimation of wheat, a process by which plants adjust Rubisco activity, the enzyme being involved in CO_2_ fixation [31]. A significantly increased C/N ratio in rice and wheat plants at e[CO_2_] were observed as compared with ambient [CO_2_] [32]. Even though there was no increase in leaf C/N ratio induced by e[CO_2_], reduced water availability increased the C/N ratio in ‘Paragon’ at 18 °C regardless of [CO_2_] and in ‘Gladius’ regardless of temperature and [CO_2_] (Figure 3). A significant increased leaf C/N under reduced water as compared with full water indicated the increased availability of carbon and reduced availability of nitrogen in wheat with less irrigation. The reduced water could decrease the content of N being absorbed by roots, resulting in less N being transported to leaves and higher leaf C/N ratio. The increased C/N ratio in ‘Gladius’ by reduced water could also be explained by the higher or non-significantly changed P_n_ in plants at RW as compared with FW (Figure 4). 

### 4.2. Genotype-Dependent Interactive Effects on Wheats with Different Heat Susceptibilities

Water, temperature, and [CO_2_] have significant individual and interactive effects on both wheat genotypes (Table 1 and Table 2). The interactive effects of multiple stresses could be antagonistic, synergistic, and additive, which corresponds to less, more, and equal effects as compared with the sum of effects of single stressors, respectively [33]. In the current study, the interactive effects of water regime, temperature, and [CO_2_] on wheat were complex and genotype dependent; however they cannot be simply concluded to be individual antagonistic, synergistic, and additive effects. For instance, the P_n_ in ‘Paragon’ at 18 °C + RW was low at both a[CO_2_] and e[CO_2_] as compared with the other three treatments, while that in ‘Gladius’ was not. Moreover, at e[CO_2_], the water regime induced an opposite effect on the photosynthetic temperature response curve of the two cultivars. 

Wheat genotypic variation could benefit our understanding of genotype by environment interaction, as genotypes with high physiological and phenotypic plasticity might have greater resilience against climatic changes [34]. A previous study showed that the acclimation strategies of ‘Paragon’ and ‘Gladius’ to climate factors varied significantly, as indicated by the developmental rate, physiological response pattern, and parameters responsible for most of the variation [34]. In accordance, the current study shows that the physiological response of wheat to temperature changes, water regime, and [CO_2_] is also genotype dependent, as shown by not only temperature response curves but also the WUE_leaf_, T_opt_, leaf C/N ratio, and pigment content. More significant differences in carotenoids between the eight treatments caused by environmental changes were observed in heat-tolerant ‘Gladius’ than heat-susceptible ‘Paragon’ (Table 2). Moreover, not only the plant genotypes will alter the effects of multiple stresses; the type, intensity, start time, and duration of each stress will also have a significant influence when two or more stresses happen simultaneously [33,35,36]. Thereby, different studies might obtain distinct conclusions on the influences of multiple stresses due to experimental design.

**Figure 7:**
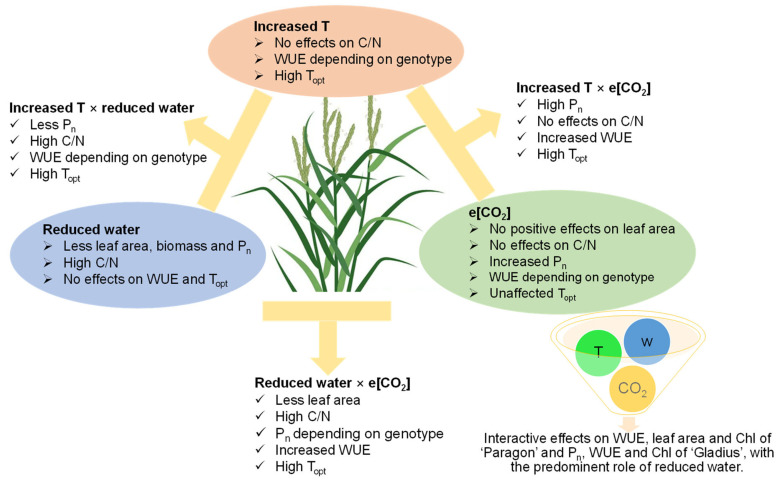
The single and interactive effects of increased temperature, reduced water, and elevated [CO_2_] on wheat physiology.

The two wheat genotypes showed similar physiological responses, but with some differences. Single factors made some significant effects on wheat physiology, while there were interactions of two or three factors (Figure 7). Together with our results that both two genotypes were clustered firstly based on water regimes from Eller et al. (2020) [34], reduced water played a predominant role among the three environmental factors in the current study (Figure 7). The interactive effects of three key environmental factors on wheat depending on genotype were complicated, which cannot be deducted from the effects of a single factor. Overall, photosynthesis was generally increased by e[CO_2_] being accompanied by more fluctuations when the plants were treated by different water regimes and temperatures. The T_opt_ was up-regulated when the growth temperature was high, and the leaf C/N ratio was increased by reduced water. The growth of both wheat genotypes was linked to the climatic effects on photosynthesis being affected by the temperature, water regime, and [CO_2_].

## Figures and Tables

**Table 1:** Output of the three-way ANOVA for net photosynthetic rate (P_n_), water use efficiency (WUE), optimum temperature (T_opt_), integrated carbon gain (∫ P_n_), leaf area, carbon/nitrogen ratio in leaves (C:N), chlorophyll a (Chl a), chlorophyll b (Chl b), carotenoids content (Car), and ratios of chlorophylls a:b (Chl a:b) and total chlorophylls to carotenoids (Chl:Car) at each temperature point of two wheat cultivars, ‘Gladius’ and ‘Paragon’.

Source of Variation	P_n15_	P_n18_	P_n25_	P_n28_	P_n35_	P_n40_	WUE_15_	WUE_18_	WUE_25_	WUE_28_	WUE_35_	WUE_40_	T_opt_	∫ P_n_	Leaf Area	C:N	Chl a	Chl b	Car	Chl a:b	Chl:Car
	‘Paragon’																				
CO_2_ × water × temp.	ns	ns	ns	ns	ns	ns	*	ns	ns	*	ns	ns	ns	ns	*	-	*	*	ns	*	*
Water × temp.	*	*	*	ns	ns	ns	ns	ns	ns	ns	ns	ns	ns	ns	***	-	*	*	ns	ns	ns
Temp. × CO_2_	ns	ns	ns	ns	ns	*	ns	*	ns	*	*	ns	ns	ns	ns	-	**	**	**	ns	**
Water × CO_2_	*	*	*	ns	ns	ns	**	ns	ns	ns	ns	ns	**	ns	ns	-	***	ns	ns	***	ns
Water	***	***	**	*	ns	ns	*	ns	ns	ns	ns	ns	**	*	***	-	*	***	*	***	***
Temp.	ns	ns	ns	ns	**	***	*	***	***	***	**	ns	***	*	**	-	ns	ns	*	**	*
CO_2_	*	**	***	***	***	***	***	***	***	***	***	***	***	***	**	-	*	ns	ns	***	ns
	‘Gladius’																				
CO_2_ × water × temp.	-	*	ns	ns	-	ns	*	ns	**	**	**	*	ns	-	ns	ns	**	***	***	***	***
Water × temp.	-	ns	ns	ns	-	ns	ns	ns	ns	ns	ns	ns	ns	-	***	**	***	***	***	***	***
Temp. × CO_2_	-	**	*	*	-	*	ns	ns	ns	ns	ns	*	ns	-	**	ns	*	ns	ns	ns	ns
Water × CO_2_	-	ns	ns	ns	-	ns	ns	ns	ns	*	ns	***	***	-	ns	ns	***	**	**	ns	*
Water	-	ns	*	ns	-	***	*	ns	*	*	ns	*	ns	-	***	***	***	**	***	ns	**
Temp.	-	ns	ns	ns	-	**	**	***	**	ns	ns	ns	***	-	***	ns	ns	ns	*	***	*
CO_2_	-	*	***	***	-	***	*	***	***	***	***	***	***	-	**	ns	ns	ns	ns	***	ns

Note: Level of significance of each factor is indicated as * *p* < 0.05, ** *p* < 0.01, *** *p* < 0.0001 within each cultivar; ‘ns’ indicates no statistically significant difference; ‘-‘ indicates parameters that did not fulfil the assumption of the parametric method. ‘Temp.’ is the abbreviation for temperature.

**Table 2:** Pigment content of wheat leaves.

	Chl *a* (mg/g DW)	Chl *b* (mg/g DW)	Car (mg/g DW)	Chl *a:b*	Chl:Car
	‘Paragon’				
a[CO_2_]					
18 °C + FW	12.49 ± 0.63 a	3.34 ± 0.20 ab	2.64 ± 0.14 a	3.74 ± 0.04 c	6.00 ± 0.10 a
18 °C + RW	10.57 ± 0.51 abc	2.23 ± 0.13 cd	2.25 ± 0.12 ab	4.76 ± 0.07 a	5.69 ± 0.07 a
28 °C + FW	9.26 ± 0.83 bcd	2.58 ± 0.28 bcd	2.25 ± 0.15 ab	3.64 ± 0.10 c	5.19 ± 0.19 b
28 °C + RW	11.94 ± 0.84 ab	2.79 ± 0.26 abc	2.49 ± 0.19 a	4.30 ± 0.08 b	5.91 ± 0.04 a
e[CO_2_]					
18 °C + FW	10.21 ± 0.58 abc	2.78 ± 0.17 abc	2.15 ± 0.13 ab	3.67 ± 0.04 c	6.05 ± 0.06 a
18 °C + RW	6.52 ± 0.78 d	1.82 ± 0.24 d	1.70 ± 0.19 b	3.61 ± 0.07 cd	4.84 ± 0.07 b
28 °C + FW	12.06 ± 0.41 ab	3.59 ± 0.12 a	2.75 ± 0.07 a	3.35 ± 0.01 d	5.68 ± 0.09 a
28 °C + RW	8.76 ± 0.62 cd	2.60 ± 0.17 bcd	2.27 ± 0.01 ab	3.35 ± 0.02 d	4.99 ± 0.04 b
	‘Gladius’				
a[CO_2_]					
18 °C + FW	10.75 ± 0.41 b	2.32 ± 0.14 bc	2.34 ± 0.07 bc	4.63 ± 0.05 a	5.57 ± 0.10 a
18 °C + RW	9.98 ± 0.73 ab	2.65 ± 0.17 c	2.35 ± 0.14 bc	3.73 ± 0.07 b	5.31 ± 0.11 bc
28 °C + FW	5.55 ± 0.55 c	1.44 ± 0.17 d	1.71 ± 0.09 d	3.89 ± 0.07 b	4.01 ± 0.20 d
28 °C + RW	13.29 ± 0.83 a	3.94 ± 0.24 a	3.27 ± 0.19 a	3.37 ± 0.04 c	5.26 ± 0.04 c
e[CO_2_]					
18 °C + FW	9.63 ± 0.80 b	2.57 ± 0.20 c	2.25 ± 0.11 c	3.73 ± 0.05 b	5.37 ± 0.18 ab
18 °C + RW	9.52 ± 0.69 b	2.58 ± 0.19 c	2.33 ± 0.12 bc	3.69 ± 0.04 b	5.15 ± 0.12 c
28 °C + FW	10.06 ± 0.28 b	2.94 ± 0.13 bc	2.57 ± 0.04 bc	3.43 ± 0.07 c	5.05 ± 0.08 c
28 °C + RW	11.42 ± 0.54 ab	3.37 ± 0.15 ab	2.81 ± 0.12 ab	3.28 ± 0.02 c	5.25 ± 0.02 c

Note: chlorophyll a (Chl a), chlorophyll b (Chl b), carotenoids content (Car), and ratios of chlorophylls a:b (Chl a:b) and total chlorophylls to carotenoids (Chl:Car). Different lowercase letters show significant difference (post hoc adjusted Bonferroni test, *p* < 0.05) between treatments within each cultivar. Data represent mean values ± S.E.M. (n = 6).

## Data Availability

Data was contained within the article.
